# Validity of the QUADAS-2 in Assessing Risk of Bias in Alzheimer's Disease Diagnostic Accuracy Studies

**DOI:** 10.3389/fpsyt.2018.00221

**Published:** 2018-05-25

**Authors:** Alisson Venazzi, Walter Swardfager, Benjamin Lam, José de Oliveira Siqueira, Nathan Herrmann, Hugo Cogo-Moreira

**Affiliations:** ^1^Department of Psychiatry and Medical Psychology, Federal University of São Paulo, São Paulo, Brazil; ^2^Department of Pharmacology & Toxicology, University of Toronto, Toronto, ON, Canada; ^3^Hurvitz Brain Sciences Program, Sunnybrook Research Institute, Toronto, ON, Canada; ^4^L.C. Campbell Cognitive Neurology Research Unit, Sunnybrook Health Sciences Centre, University of Toronto, Toronto, ON, Canada; ^5^Brain Sciences Research Program, Sunnybrook Research Institute, University of Toronto, Toronto, ON, Canada; ^6^Division of Neurology, Department of Medicine, University of Toronto, Toronto, ON, Canada; ^7^Institute of Psychology, São Paulo University, São Paulo, Brazil; ^8^Hurvitz Brain Sciences Research Program Sunnybrook Health Sciences Centre, University of Toronto, Toronto, ON, Canada; ^9^Division of Geriatric Psychiatry Sunnybrook Health Sciences Centre, Toronto, ON, Canada; ^10^Department of Psychiatry and Medical Psychology, Federal University of São Paulo, São Paulo, Brazil; ^11^Laboratory of Innovation in Psychometrics (LIP), São Paulo, Brazil

**Keywords:** Alzheimer, diagnosis, scale evaluation, psychometrics, biostatistics

## Abstract

Accurate detection of Alzheimer's disease (AD) is of considerable clinical importance. The Quality Assessment of Diagnostic Accuracy Studies 2 (QUADAS-2) is the current research standard for evaluating the quality of studies that validate diagnostic tests; however, its own construct validity has not yet been evaluated empirically. Our aim was to evaluate how well the proposed QUADAS-2 items and its domains converge to indicate the study quality criteria. This study applies confirmatory factor analysis to determine whether a measurement model would be consistent with meta-analytic data. Cochrane meta-analyses assessing the accuracy of AD diagnostic tests were identified. The seven ordinal QUADAS-2 items, intended to inform study quality based on risk of bias and applicability concerns, were extracted for each of the included studies. The QUADAS-2 pre-specified factor structure (i.e., four domains assessed in terms of risk of bias and applicability concerns) was not testable. An alternative model based on two correlated factors (i.e., risk of bias and applicability concerns) returned a poor fit model. Poor factor loadings were obtained, indicating that we cannot provide evidence that the indicators convergent validity markers in the context of AD diagnostic accuracy metanalyses, where normally the sample size is low (around 60 primary included studies). A Monte Carlo simulation suggested that such a model would require at least 90 primary studies to estimate these parameters with 80% power. The reliability of the QUADAS-2 items to inform a measurement model for study quality remains unconfirmed. Considerations for conceptualizing such a tool are discussed.

## Introduction

Alzheimer's disease (AD), singly or in combination with other neuropathological processes, is responsible for the majority of dementia cases worldwide. In part because of its frequent co-occurrence with other conditions (1), and its own marked phenotypic variability (2), precise diagnosis remains challenging (3). Significant progress has been made in the development of AD biomarkers, including medial temporal lobe atrophy on magnetic resonance imaging (MRI) (4, 5), temporoparietal hypometabolism or hypoperfusion on positron emission tomography (PET) (6, 7), alterations in cerebrospinal fluid amyloid, tau, and phosphorylated tau levels (8), amyloid-ligand PET (9), and most recently tau-ligand PET (10, 11). Despite these advances, diagnosis remains reliant on clinical assessment. Biomarkers are supportive, rather than diagnostic, and their incorporation into the newest generation of diagnostic criteria has been inconsistent.

The National Institute of Neurological Disorders and Stroke–Alzheimer Disease and Related Disorders (NINCDS–ADRDA) criteria (12), served as the research standard until it was superseded by the National Institute on Aging—Alzheimer's Association (NIA-AA) (13). Its companion criteria was the Diagnostic and Statistical Manual of Mental Disorders, fourth edition (DSM-IV-TR) (14), itself recently revised in the Diagnostic and Statistical Manual of Mental Disorders, fifth edition (DSM-V) (15). Although not formally designed for clinical use, both have heavily informed the medical diagnosis of AD. They have since been joined by the International Working Group (IWG) criteria (16–18). The NIA-AA uses biomarkers in a supportive role, the DSM-V does not require them at all, and the IWG considers them mandatory. These differences in approach reflect lingering uncertainty regarding the validity of AD diagnostic tests. However, diagnosis must move beyond clinical features alone in order to provide a more cogent linkage between nosology and biological mechanisms. There is therefore a crucial need for validation studies examining the accuracy of AD diagnostic tests.

When examining diagnostic accuracy studies, it is important to discriminate between the accuracy of the proposed diagnostic test, and any methodological issues that could inflate or underestimate the reported results, including uniform assessment of study quality (19). The Quality Assessment of Diagnostic Accuracy Studies (QUADAS) was developed specifically to assess the methodological rigor of diagnostic accuracy studies in systematic reviews (20).

The QUADAS was conceived in 2003, by a panel of nine experts in the field of diagnostics that, using a Delphi procedure (21), who evaluated 55 studies investigating the effects of bias and variation on measures of test performance. It was considered that sources of bias best supported by empirical evidence were: variation by clinical and demographic subgroups, disease prevalence/severity, partial verification bias, clinical review bias and observer/instrument variation (22). Initially a list of 28 items (22, 23) was produced, which was later reduced to 14 items in a Likert scale format with three categories of answers (high risk, unclear, low risk). A revised scale, the QUADAS-2, was proposed in 2011 to “measure the degree to which individual study criteria match the review question” (24), which includes seven of the original 14 items. At that time, the authors emphasized that further research would be necessary to determine the usability and validity of the instrument (22).

Since 2011, the QUADAS-2 has been adopted widely and applied in reviews of diagnostic accuracy studies across many different medical areas, raising some concerning questions regarding QUADAS-2 by some authors. Schueler et al. (25) indicated a limitation associated with calculating inter-rater agreement only on the domain questions. Cook et al. (24) felt that the tool was not able to discriminate between poorly and strongly designed studies, and that the QUADAS-2 offered no obvious advantage over to the original 14-item QUADAS. Other authors have criticized the purposively qualitative nature of the QUADAS-2, which does not recommend scoring a study using a numeric value, a fundamental quality of assessment scales (24).

Because the QUADAS-2 proposes to assess quality using observed items, it is important to consider not only the validity of those items (i.e., content validity) and additionally, whether the items inform an underlying construct (i.e., construct validity). The seven QUADAS-2 items were designed to assess the risk of bias associated with, and/or the applicability to the general population of, four methodological points (patient selection, the index test, the reference standard used, and the flow of patients through the study or timing of the index test and reference standard) (25). Although all seven items have content validity (26–28), their validity to inform the underlying construct of quality has not been tested. This type of validity is tested empirically to determine if the items function as reliable indicators of their supposed underlying constructs (29). If the indicators cannot be assessed reliably between studies, the perceived quality of evidence may be inaccurate. Therefore, it remains to be determined, in a practical sense, whether the QUADAS-2 items, individually or taken as a whole, offer a valid measurement of methodological quality in studies of diagnostic tests for AD.

Confirmatory factor analysis (CFA) is an indispensable analytic tool for construct validation (also called factorial validity or internal consistency) (30). The technique is ideally suited to determine how well each of the seven items measure the two proposed domains (i.e., Risk of Bias and Applicability Concern). CFA might be used to evaluate how well the proposed items and domains converge to indicate the study quality criteria (i.e., convergent validity). This study applies CFA to determine whether a two-factor factor model (bias, application) for the QUADAS-2 is consistent with the (meta-analytic) data in AD diagnostic accuracy studies.

## Methods

This study was approved by the Ethics Committee of Research of the Federal University of São Paulo (UNIFESP) under protocol number 2613240615. The Cochrane Library was searched for (1) meta-analyses of (2) diagnostic accuracy studies where (3) the subject was AD. Studies reporting on other types of Dementia and Cognitive impairment were excluded. Primary studies that were assessed using the QUADAS-2 were identified and any duplicate primary study entries across the meta-analyses were removed. The Reviewers' assessments of each of the seven QUADAS-2 items were recorded.

**Figure 1:**
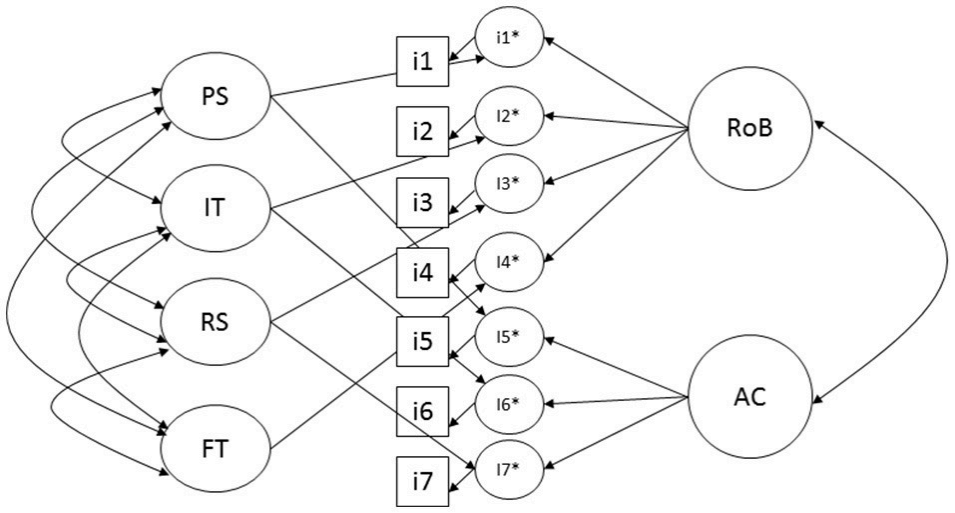
Multitrait-multimethod conceptual model for QUADAS-2. RoB, risk of bias; AC, applicability concern; PS, patient selection; IT, index test; RS, reference standard; FT, flow and timing.

**Figure 2:**
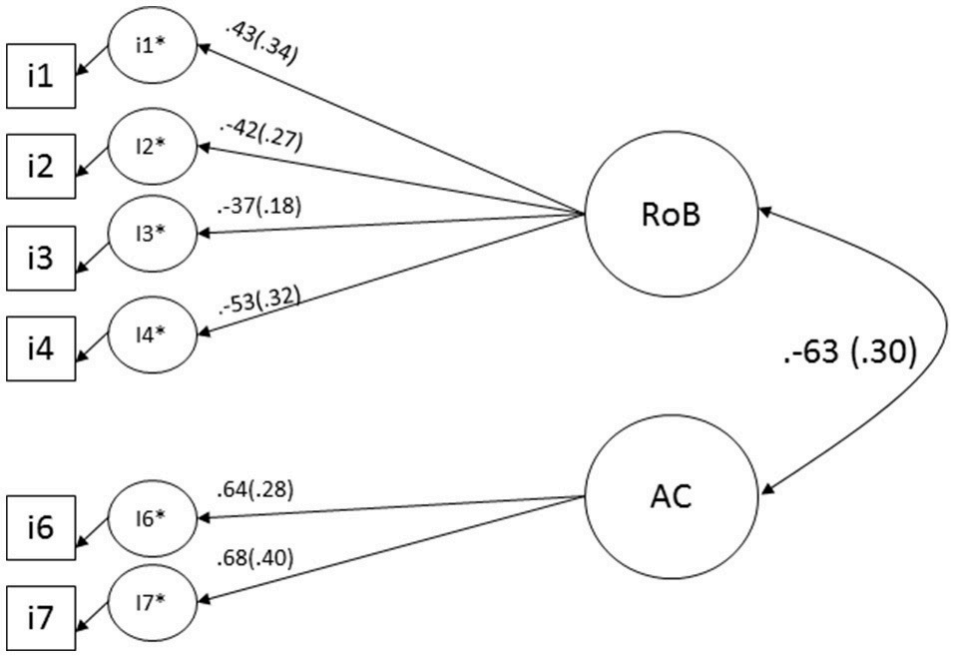
Correlated-factor model for QUADAS-2 with standardized factor loadings and standard errors in parenthesis. RoB, risk of bias; AC, applicability concern.

CFA, a structural equation modeling technique, was used to evaluate the construct validity of QUADAS-2. As previously defined Bollen (29), p. 182, “a measurement model specifies a structural model connecting latent variables to one or more measures or observed variables” (also called indicators, represented by squares in Figures 1, 2). Domains are latent variables not directly observed (represented by ovals/circles) but rather informed by the observed indicators. In the context of structural equation modeling (a statistical technique which deals with non-observed phenomenon), the risks of bias might not be measured directly and therefore are called latent. In other words, a construct or latent (in this case, risk of bias) represents what is common within observable variables the seven criteria used by Cochrane to measure bias.

The application of CFA assumes that studies have an underlying intrinsic quality, and that this quality causes the studies to have more favorable design and reporting characteristics. This representation of a latent phenomenon is called a reflective model. In contrast, a formative model would characterize the studies by multiple markers of quality that may be correlated but not necessarily causally related to each other or to an underlying attribute, which together could be used to summarize aggregate quality. Formative models, in which a composite variable is modeled as weighted sum of the item scores [see (31) for an introduction to formative versus reflective models], have specific requirements for the identification of its measurement models but, if met, then a formative model would be identified. Some authors describe formative models as *hardy to identify* [for major details see (32)]. Moreover, because cause indicators are exogenous, their variances and covariances are not explained by a formative measurement model, which makes it more difficult to assess the validity of a set of cause indicators (29). Here we use a reflective model to explicitly test whether the items inform the underlying latent construct of study quality.

Following the theoretical definition given by the Cochrane Collaboration that defines quality as “both the risk of bias and applicability of a study” (20) and the assertion that the QUADAS-2 that “…comprises four domains: patient selection, index test, reference standard, and flow and timing. Each domain is assessed in terms of risk of bias, and the first 3 domains are also assessed in terms of concerns regarding applicability” (20), hence, a multitrait-multimethod CFA could reproduce the above description in term of CFA. Another more parsimonious way to transpose the QUADAS-2 description in terms of CFA's models is with only two factors. Such a solution might be reasonably evaluated due to identification rules below described.

### Sample size and heterogeneity

To conduct CFA, our sample size constituted 58 primary accuracy studies within the five following systematic reviews (33–37), included primary accuracy studies from 1946 to 2013. The systematic reviews aimed to determine the diagnostic accuracies (from neuropsychological tests to biomarkers as PET imaging with the ^11^C-labeled Pittsburgh Compound-B and cerebrospinal fluid). No language or date restrictions were applied to the electronic searches and methodological filters used in the systematic reviews, maximizing sensitivity and given heterogeneity to the sampling. There was no selection process specific to AD instruments, using all the available systematic reviews from Cochrane Library. The tests evaluated in these five systematic reviews include the main techniques used to detect AD. Details about the limitations of QUADAS-2 use under different sample sizes in the context of systematic reviews will be discussed below in the statistical analysis subheading.

### Statistical analysis

As an initial inspection, a simple correlation between the seven items was done using a polychoric matrix; it is similar to Pearson correlation matrix, but because the QUADAS-2 items are categorical the correlation are based on polychoric point estimation.

Because the QUADAS-2 items are ordered-categorical (i.e., low risk, unclear, and high risk), the weighted least squares mean- and variance-adjusted (WLSMV) estimator was used. This estimator offers more precise estimates of the factor loadings (38) for categorical observed indicators (items), and it is the default estimator in Mplus (39). Due to the complex sampling structure (i.e., 58 original accuracy studies nested in five systematic reviews), standard errors were computed by a sandwich estimator and chi-square test of the model fit took into account the non-independence of observation; for major details and discussion about such implementation see (40, 41). The adopted statistical significance level was 0.05.

The following fit indices were used evaluate the model fit for CFA: chi-square, comparative fit index (CFI), Tucker-Lewis Index (TLI), root mean square error of approximation (RMSEA), and weighted root mean square residual (WRMR). For both the CFI and TLI, values >0.90 and 0.95 were considered acceptable and optimal fits to the data, respectively. For the RMSEA, values <0.06 were considered reasonable and optimal fit to the data, respectively. For WRMR, values near or below 0.90 were considered adequate (42). To evaluate the magnitude of correlation between the latent response variables for QUADAS-2 items and the factors, we used the factor loadings. To overcome the disadvantages of Cronbach's alpha (43), scale reliability for QUADAS-2 model was estimated via factor loadings of CFA as described by Jöreskog (44).

Lastly, based on the obtained estimates (e.g., factor loadings reported in the Figure 2), we conducted a Monte Carlo simulation analysis to evaluate the power and other related parameters for different sample sizes of meta-analyses. Ten thousand replications were considered to ensure the stability of the results (e.g., average of the parameter estimates across replications). The following criteria, as described by Muthén and Muthén (45), were considered for the evaluation of the adequacy of the sample size: (1) the proportion of replications for which the 95% confidence interval contains the true population parameter value should between 0.91 and 0.98 and (2) the power for each parameter must be superior to 0.80, as largely used (46). The analysis were implemented in Mplus version 8.0.

## Results

Table 1 shows the proportions and counts for the seven ordinal items of QUADAS-2.

**Table 1:** Proportions and counts for the seven items of QUADAS-2 (*n* = 58).

**Items**	**Answers**	**Proportions**	**Counts**
Item 1 (Risk of bias: patient selection)	High risk	0.224	13.000
	Unclear	0.448	26.000
	Low risk	0.328	19.000
Item 2 (Risk of bias: inex test)	High risk	0.466	27.000
	Unclear	0.121	7.000
	Low risk	0.414	24.000
Item 3 (Risk of bias: reference standard)	High risk	0.103	6.000
	Unclear	0.552	32.000
	Low risk	0.345	20.000
Item 4 (Risk of bias: flow and timing)	High risk	0.241	14.000
	Unclear	0.172	10.000
	Low risk	0.586	34.000
Item 5 (Applicability concerns: patient selection)	Unclear	0.069	4.000
	Low risk	0.931	54.000
Item 6 (Applicability concerns: index test)	Unclear	0.121	7.000
	Low risk	0.879	51.000
Item (Applicability concerns: reference standard)	High risk	0.052	3.000
	Unclear	0.086	5.000
	Low risk	0.862	50.000

### Testing QUADAS-2 models: multitrait-multimethod model

The four domains representation of QUADAS-2's structure might be depicted by multitrait-multimethod model (Figure 1), where at the same time there are the four groups of items and concomitantly they are measuring risk of bias and applicability concerns. However, such a model structure is not identified, because models with more than one domain must have at least two indicators per domain (29, 47, 48). This limitation of the QUADAS-2 precludes assessment under such methodology.

### Two correlated factors model

An alternative representation, a two correlated factors model (Figure 2) is testable; however, Item 5 (Applicability concerns related to Patient Selection) was almost perfectly correlated (polychoric correlation = −0.987) with Item 7 (Applicability Concerns related to the Reference Standard). This occurs due to bivariate empty cells (i.e., zero values in a 3 × 3 cross tab between some pairs of items in the polychoric correlation matrix) and as consequence such a solution (the seven indicators together) for QUADAS-2 is inadmissible; therefore, we tested a reduced version without one of the QUADAS items involved in the high correlation.

Removing Item 5, the following fit indices were obtained [χ^2^_(8)_ = 7.883, *p* = 0.445, CFI of 1.00, TLI of 1.044, RMSEA of 0.000 and WRMR of 0.493; Figure 2].

A naïve interpretation of above fit indices would conclude that the model has excellent fit indices. However, a model cannot be retained based solely on values of global fit statistics; the residuals, such as standardized, normalized, correlation, or covariance residuals, must also be considered. The magnitudes of the factor loadings were very low (Figure 2). Examining the correlations among the individual items (Table 2), with the exception of the correlation between Item 3 (Risk of Bias of Reference Standard) and Item 7 (Applicability Concerns of Reference Standard), the correlations between the items were also very low. As mentioned above, it is equally important to consider the size of the model's parameter estimates as it is to consider the model goodness of the fit in determining the factor load (30).

**Table 2A:** Polychoric correlation matrix (standard errors in parenthesis).

	**Item 1**	**Item2**	**Item 3**	**Item 4**	**Item 5**	**Item 6**	**Item 7**
Item 1	1						
Item 2	−0.301 (0.15)	1					
Item 3	−0.022 (0.17)	0.107 (0.18)	1				
Item 4	−0.270 (0.15)	0.217 (0.17)	0.214 (0.18)	1			
Item 5	0.096 (1.00)	−0.038 (0.38)	−0.262 (0.33)	0.117 (0.48)	1		
Item 6	−0.313 (0.30)	0.265 (0.37)	0.107 (0.24)	0.202 (0.31)	0.611 (0.25)	1	
Item 7	−0.054 (0.20)	0.421 (0.22)	0.661 (0.19)	0.117 (0.25)	−0.987 (0.00)	0.437 (0.26)	1

*Item 1, Risk of bias-patient selection; Item 2, Risk of bias-index test; Item 3, Risk of bias-reference standard; Item 4, Risk of bias-flow and timing; Item 6, Applicability concerns-index Test; Item 7, Applicability concerns-reference standard*.

As a result of poor correlations, presented in Table 2A, the residual variances are greater than the common variances. Residual variance is that unexplained by the factor that the indicator is supposed to measure. Table 2B shows the model estimated correlation residual, where these residuals exceed 0.10 in absolute value. Thus, the model does not explain very well the observed correlation between their variables; specifically, the model underpredicts their association.

**Table 2B:** Model estimated residual correlation.

	**Item 1**	**Item 2**	**Item 3**	**Item 4**	**Item 6**	**Item 7**
Item 1						
Item 2	−0.180					
Item 3	−0.158	0.156				
Item 4	−0.218	0.216	0.190			
Item 6	−0.172	0.170	0.149	0.207		
Item 7	−0.184	0.182	0.160	0.222	0.437	

*Item 1, Risk of bias-patient selection; Item 2, Risk of bias-index test; Item 3, Risk of bias-reference standard; Item 4, Risk of bias-flow and timing; Item 6, Applicability concerns-index Test; Item 7, Applicability concerns-reference standard*.

Standardized factor loadings are also a proxy of item reliability, where a higher factor loading indicates a more reliable item. The reliability for risk of bias factor is 0.40 and for applicability concern is 0.28.

Considering low factor loadings and high residual variances, we are forced to conclude a lack of evidence to support that the QUADAS-2 items adequately inform the measurement model underlying the included studies.

The results of a Monte Carlo simulation, for different sample sizes of systematic reviews, are presented in Table 4, where only systematic reviews with more than 90 primary studies would offer power higher 0.8 for the majority of the items. The exception is item 3 (risk of bias: reference standard) which would have a power of 0.722 given 90 included primary studies.

## Discussion

This study offers some evidence evaluating the construct validity of the QUADAS-2 for assessing the quality of studies supporting AD diagnostic tests. Although fit indices (CFI, TLI, RMSEA) were adequate, it is a poor practice to decide on whether to retain a model based solely on values of global fit statistics instead of also considering the residuals, such as standardized, normalized, correlation, or covariance residuals. This is because poor model fit at the level of the residuals is not always detected by global fit statistics [e.g., (49, 50)].

The original QUADAS-2 tool included 4 key domains that pertain to patient selection, index test, reference standard, and flow and timing (i.e., flow of patients through the study and timing of the index tests and reference standard).

In order to create a testable measurement model in the context of CFA, we transposed the QUADAS-2 items into two-correlated factor solution (Figure 2). Although the originally proposed four factors might be better described using a multitrait-multimethod model, or even with four-correlated factor solution, statistically those models would be inadmissible due to the limited number of items per factor (at least two items per factor would be necessary (29, 47, 48). Therefore, in order to produce a “testable” model that could provide evidence supporting a four-factor tool, additional indicators would be required. However, the alternative measurement model, consisting of two domains informed by seven indicators, was testable after removing a redundant Applicability Concern item. The obtained fit indices support the validity of this model.

Although we tested a model that assumes that the QUADAS-2 items inform an underlying construct of study quality, the QUADAS-2 might also be considered “critical reading grid” to which construct validity might not apply. We argue against this notion for two reasons. First, here we provide empirical support that the items do inform such an underlying construct. Second, it is clear that throughout the development of the QUADAS-2, a theory about what items would be used to evaluate bias/applicability, and how those items would be grouped indicated the structure of the model *a priori*. Although a negative result would not necessarily bring into question the practical utility of the tool, or the validity of its content, as a model, the QUADAS-2 is liable to be tested.

Despite the reduction of QUADAS items from 28 to 7 since its inception, the empirical evidence provided here, suggests that two of the extant items remain redundant when assessing the AD literature. This is particularly problematic because assessors may be unduly biased if a single underlying quality feature is represented by 2 out of 7 items. The reduction of QUADAS items for the QUADAS-2 was performed using a Delphi procedure (51), which is not based on CFA, or grounded in item response theory, which are preferred methods to evaluate construct validity (52) and items selection. A re-examination of the QUADAS-2 items based on modern item response theory might allow for an improved measurement model.

The items were poorly correlated and possibly unreliable (Tables 2A,B) because residual variances were high (Table 3). Residual variance is the variance unexplained by the factor that the indicator is intended to measure. A small N and a small number of items per factor likely contributed to the high relative percentage bias estimated under WLSMV. Due to these considerations, some susceptibility to random measurement error might have been expected; however, if the QUADAS-2 items had been more closely related to their underlying factors, more precise estimates would have been possible [(50), p. 9–10].

**Table 3:** Common variance, its standard errors, *p*-values, and residual variance for each QUADAS-2 Items.

**Indicators**	**Common variances**	**S.E**.	***P*-value**	**Residual variance**
ITEM1	0.182	0.293	0.535	0.818
ITEM2	0.178	0.230	0.439	0.822
ITEM3	0.137	0.137	0.317	0.863
ITEM4	0.263	0.326	0.420	0.737
ITEM6	0.408	0.357	0.253	0.592
ITEM7	0.468	0.546	0.391	0.532

*S.E, Standard error; Item 1, Risk of bias-patient selection; Item 2, Risk of bias-index test; Item 3, Risk of bias-reference standard; Item 4, Risk of bias-flow and timing; Item 6, Applicability concerns-index test; Item 7, Applicability concerns-reference standard*.

**Table 4:** Monte carlo simulation study evaluating the proportion of replication and power for different meta-analysis sample size.

**Item/Factor correlation**	***N* = 60**		***N* = 60**		***N* = 60**		***N* = 60**		***N* = 60**		***N* = 60**		***N* = 60**	
	**PR**	**Power**	**PR**	**Power**	**PR**	**Power**	**PR**	**Power**	**PR**	**Power**	**PR**	**Power**	**PR**	**Power**
Item1	0.936	0.699	0.941	0.700	0.932	0.754	0.918	0.782	0.934	0.789	0.933	0.828	0.921	0.827
Item 2	0.934	0.660	0.922	0.693	0.932	0.727	0.936	0.754	0.936	0.793	0.924	0.810	0.942	0.819
Item 3	0.947	0.553	0.918	0.590	0.940	0.613	0.938	0.643	0.914	0.659	0.918	0.699	0.927	0.722
Item 4	0.910	0.754	0.903	0.773	0.915	0.816	0.926	0.819	0.916	0.858	0.920	0.898	0.927	0.915
Item 6	0.962	0.848	0.956	0.857	0.952	0.865	0.951	0.889	0.947	0.922	0.965	0.916	0.968	0.938
Item 7	0.966	0.865	0.975	0.878	0.975	0.880	0.957	0.885	0.973	0.930	0.961	0.928	0.982	0.940
Correlation between the factors	0.962	0.744	0.968	0.771	0.961	0.789	0.963	0.817	0.961	0.864	0.953	0.863	0.942	0.869

*PR, Proportion of replication*.

The poor factor loadings observed have important implications for bias assessment in systematic reviews. Here we were unable to provide evidence that the indicators have convergent validity of the intended domains. In part, this is because the sample size (i.e., the number of included primary studies in most systematic reviews) was too low for the factor loadings to be estimated properly [see (53)]. In practice, this means that the generalizability of the clinical findings was not explicitly evaluable. In our Monte Carlo simulation, the number of primary studies that would be necessary to achieve a power of 0.8 was at least 90, given the data in this systematic review. Because large sample sizes are not often available, it might be useful to redefine some QUADAS-2 indicators with the intention that they should be more strongly related to the underlying domains.

The structure of the QUADAS-2 hindered testing its measurement model; the tool might be augmented with more indicators per factor. Because the proposed structure of the model, untestable in a multitrait-multimethod model with four domains, necessitated transposition into a two-factor oblique model which may have introduced a loss of information. Nonetheless, these considerations do not affect the model fit indices, which offer some evidence of construct validity.

In the case of the first three QUADAS-2 items (residual variances 0.818, 0.822, and 0.863), nearly the entire factor was explained by residual variance, which is usually due to random measurement error and/or rater unreliability. The relevance of these items as indicators of study quality in AD was not empirically supported. Among the Applicability Concern items, the best indicator explained 46% of the common variance, still less than the residual variance (i.e., 54%). Given the importance of the parameter estimates (Brown, p. 135–136) as criteria for the utility of a scale, and the unacceptably high proportion of variance due to random measurement error and/or rater unreliability, this first attempt to evaluate empirically the QUADAS-2 items to inform Risk of Bias and Applicability Concerns in the AD literature does not support their reliability.

While this study focused on AD studies, the QUADAS-2 is used to assess quality and internal validity of tests to diagnose many neurological and other disorders including other forms of dementia (54, 55), Parkinson's disease (56) and Stroke (57, 58). It is possible that some QUADAS-2 risk of bias items may be more difficult to assess or less applicable in AD studies. For instance, in the AD studies, some difficulties evaluating risk of bias related to the index test arose from signaling questions related to the use of a pre-specified cut-off. No consensus exists for 11C-PIB-PET (37), 18FDG-PET (36), CSF, and serum biomarker (35) cut-offs, partly because information has been lacking or inconsistent, and measurements vary considerably between labs. When this criterion was applied to those biomarker studies, it may have contributed to over-estimation of risk of bias. In contrast, pre-specified cut-offs could have been justified in studies evaluating the Mini Mental State Examination, and this signaling question was appropriately applied to evaluate the index test in those studies.

Throughout the meta-analyses, the majority of criteria assessed as “high” risk of bias achieved this score based on a lack of reporting, rather than a confirmed risk of bias due to inadequate study design per se. Certain metanalyses employed different thresholds to endorse high vs. unclear risk. For instance, studies evaluating the Montreal Cognitive Assessment, were judged to be of “unclear” risk of bias where reporting of pre-specified cut-offs was absent or unclear (33); however the same criteria were judged to endorse a high risk of bias among studies evaluating the Mini Mental State Examination. This discrepancy in implementation may have contributed to the compromised reliability of these items in the AD studies.

Some important limitations might be considered. First, we were only able to use meta-analyses where item-level QUADAS-2 data for each primary study was reported, resulting in some loss of data (59). Second, the agreement between the judges in the majority of the publications was not reported, limiting our ability to comment on the contribution of disagreement or differential interpretation of the seven items to lack of item reliability. Third, the sample size was limited and, therefore, the magnitudes of the factor loadings were not precisely estimated; however, the sample size is consistent with that of other systematic reviews, raising an issue about the content of the QUADAS-2 items, and its testability, in the general context of systematic reviews. Regarding the generalizability of the present findings, it should be considered that the measurement properties of the QUADAS-2 might behave differently between different diseases, or between different diagnostic procedures for the same disease (59, 60).

In conclusion, although the findings do not necessarily inform the practical utility of the scale in identifying areas of weakness within a study, evidence to support the reliability of the QUADAS-2 items to inform study quality remains lacking. Further research might evaluate whether the present findings regarding the QUADAS-2 are specific to AD studies, or generalizable to other fields of medicine. Additional empirical evidence and additional analyses based on modern item response theory would be needed in order to propose a reliable set of study quality criteria for use in AD diagnostic accuracy studies.

## Author contributions

AV, JdO, and HC-M designed and ran the analysis. WS, BL, and NH gave an important contribution to the interpretation of the results and their effect sizes applied to the clinical practice. All authors revised the last version of the manuscript.

### Conflict of interest statement

The authors declare that the research was conducted in the absence of any commercial or financial relationships that could be construed as a potential conflict of interest.
